# Insulin: too much of a good thing is bad

**DOI:** 10.1186/s12916-020-01688-6

**Published:** 2020-08-21

**Authors:** Hubert Kolb, Kerstin Kempf, Martin Röhling, Stephan Martin

**Affiliations:** 1 Faculty of Medicine, University of Duesseldorf, Moorenstr. 5, 40225 Duesseldorf, Germany; 2 West-German Centre of Diabetes and Health, Duesseldorf Catholic Hospital Group, Hohensandweg 37, 40591 Duesseldorf, Germany

**Keywords:** Hyperinsulinemia, Insulin resistance, Nrf2, Autophagy, eNOS, Obesity, Type 2 diabetes mellitus, Inflammation, Oxidative stress, Cardiovascular morbidity and mortality

## Abstract

**Background:**

Insulin shares a limited physiological concentration range with other endocrine hormones. Not only too low, but also too high systemic insulin levels are detrimental for body functions.

**Main body:**

The physiological function and clinical relevance of insulin are usually seen in association with its role in maintaining glucose homeostasis. However, insulin is an anabolic hormone which stimulates a large number of cellular responses. Not only too low, but also excess insulin concentrations are detrimental to the physiological balance. Although the glucoregulatory activity of insulin is mitigated during hyperinsulinemia by dampening the efficiency of insulin signaling (“insulin resistance”), this is not the case for most other hormonal actions of insulin, including the promotion of protein synthesis, de novo lipogenesis, and cell proliferation; the inhibition of lipolysis, of autophagy-dependent cellular turnover, and of nuclear factor E2-related factor-2 (Nrf2)-dependent antioxidative; and other defense mechanisms. Hence, there is no general insulin resistance but selective impairment of insulin signaling which causes less glucose uptake from the blood and reduced activation of endothelial NO synthase (eNOS). Because of the largely unrestricted insulin signaling, hyperinsulinemia increases the risk of obesity, type 2 diabetes, and cardiovascular disease and decreases health span and life expectancy. In epidemiological studies, high-dose insulin therapy is associated with an increased risk of cardiovascular disease. Randomized controlled trials of insulin treatment did not observe any effect on disease risk, but these trials only studied low insulin doses up to 40 IU/day. Proof for a causal link between elevated insulin levels and cardiovascular disease risk comes from Mendelian randomization studies comparing individuals with genetically controlled low or high insulin production.

**Conclusions:**

The detrimental actions of prolonged high insulin concentrations, seen also in cell culture, argue in favor of a lifestyle that limits circadian insulin levels. The health risks associated with hyperinsulinemia may have implications for treatment regimens used in type 2 diabetes.

## Background

Most endocrine hormones exhibit a window of optimal physiological concentrations, with compromised function of the organism at levels below or above that range. For instance, subnormal levels of thyroid hormone define the clinical condition of hypothyroidism, above normal levels represent hyperthyroidism which usually requires therapy. Addison’s disease is characterized by insufficient cortisol production, while excess synthesis is seen in Cushing syndrome.

For insulin, we argue here that not only hypoinsulinemia but also hyperinsulinemia is detrimental to body functions. Hypoinsulinemia causes insulin-deficient diabetes, and the hormonal actions of insulin are necessary for the life of complex organisms [1]. On the other hand, permanently elevated levels of insulin may cause disturbance of normal cellular physiology and organ function. We describe the molecular basis of these undesired insulin actions and consequences of hyperinsulinemia for health-relevant endpoints, such as obesity or cardiovascular diseases.

## Main text

### Insulin signaling pathways

**Fig. 1:**
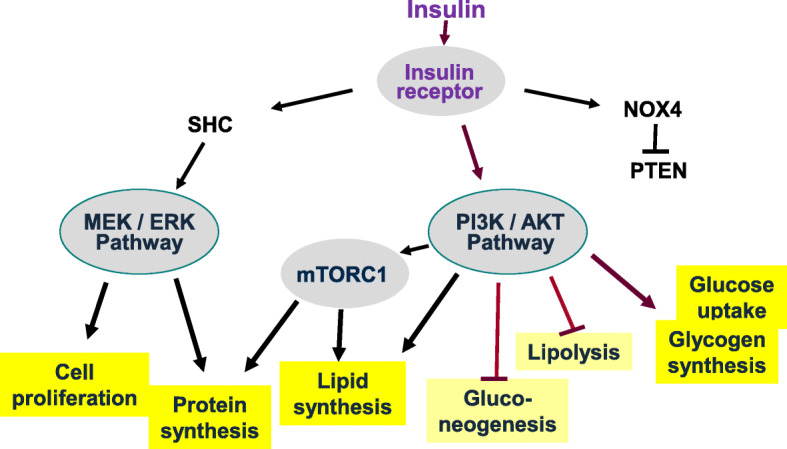
Metabolic signaling of insulin is anabolic. Insulin signaling through the insulin receptor engages several pathways and results in an anabolic state of metabolism. The canonical pathway via phosphokinases PI3K and AKT/PKB promotes glucose uptake and glycogen and lipid syntheses, whereas lipolysis is inhibited in adipocytes, as well as hepatic gluconeogenesis. In addition, AKT kinases activate mTORC1 which supports de novo lipogenesis and protein synthesis. The insulin signaling pathway via SHC and the MAP kinases MEK and ERK promotes cell proliferation and protein synthesis. Another insulin signaling pathway involves NOX4 and the inhibition of PTEN, an inhibitor of the PI3K-AKT pathway

Binding of insulin to its cognate cell surface-bound receptor causes a conformational change which initiates a cascade of signaling events. Autophosphorylation by the insulin receptor tyrosine kinase is accompanied by tyrosine phosphorylation of receptor substrates, such as insulin receptor substrate (IRS) and Src homology 2 domain-containing transforming proteins (SHC) proteins. Phosphorylation of IRS allows binding of phosphatidylinositol-3-kinase (PI3K) and synthesis of phosphatidylinositol (3,4,5)-trisphosphate (PIP_3_), which eventually leads to the phosphorylation and activation of the serine/threonine-specific protein kinase B (AKT). Upon activation, AKT interacts with several substrates which mediate anabolic effects of insulin; these include glucose uptake, glycogen synthesis, de novo lipogenesis, and protein synthesis [2]. Additional pathways triggered by the activated insulin receptor comprise phosphorylation of SHC, followed by activation of the Rat sarcoma (Ras)–rapidly accelerated fibrosarcoma (Raf)–mitogen-activated protein kinase kinase (MEK)–extracellular signal-regulated kinase (ERK) pathway. The terminal kinase ERK is a mitogen-activated kinase promoting cell proliferation and further cellular activities including protein synthesis [3]. Another pathway triggered by the engaged insulin receptor involves activation of NADPH oxidase 4 and subsequent hydrogen peroxide-mediated inhibition of phosphatase and tensin homolog (PTEN), which is an important negative regulator of PI3K signaling [4] (Fig. 1).

### Insulin secretion

Insulin secretion by pancreatic islet β cells responds to the level of circulating nutrients such as glucose, amino acids, and free fatty acids. Sweeteners may further increase carbohydrate-induced insulin secretion. A large number of endogenous factors contribute to the regulation of β cell activity, either stimulatory, inhibitory, or both context-dependent. These include hormones, neurotransmitters, and immune mediators [5–12]. Insulin is essential for maintaining glucose homeostasis, primarily by facilitating the post-meal uptake of glucose into muscle and fat cells via translocation of the glucose transporter 4 [13]. In the absence of dietary glucose supply and after depletion of glycogen stores, glucose in circulation primarily comes from gluconeogenesis in the liver. If circulating insulin levels are below the concentrations required for stimulating glucose uptake from the blood, endogenous stores of fat and protein must be used for energy production. For the maintenance of life in the fasting state, circulating insulin levels range between approx. 25 and 70 pmol/l (25–75% percentile), as determined for healthy adult persons in the *National Health and Nutrition Examination Survey* (NHANES) [14]. In response to meals with varying carbohydrate content, insulin levels may rise to the range of approx. 300–800 pmol/l [15].

### Insulin promotes obesity

**Fig. 2:**
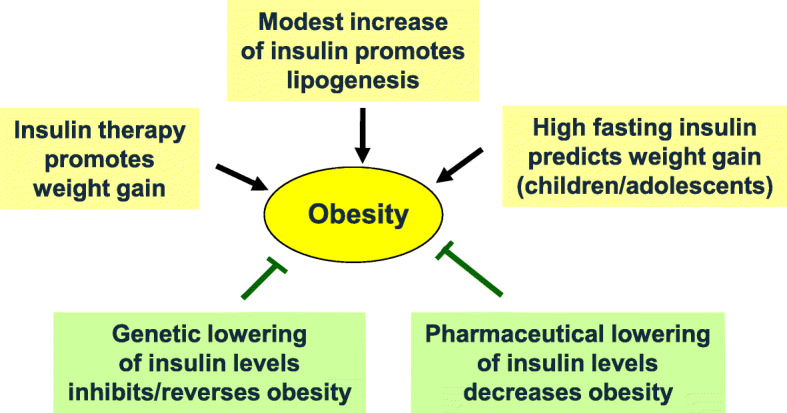
Insulin promotes obesity. Several independent types of observations support the conclusion that insulin promotes adipogenesis and obesity. For details, see description in the general text

Almost 100 years ago, insulin injections were one of the options of therapy in nondiabetic persons suffering from undernutrition in the context of various diseases. Insulin doses were in the range of those applied in type 1 diabetes and led to increased appetite and weight gain [16]. Indeed, one major function of insulin as an anabolic hormone is to favor energy storage over usage. This is reflected by the finding that insulin infusion (1 mU/kg/min) significantly inhibits lipolysis in the skeletal muscle (about 43%) and even more effective in adipose tissue (about 75%) [17]. Doubling fasting insulin levels suffices to inhibit lipolysis by approx. 50% and to promote lipogenesis (for both, mean insulin concentration for 50% effect (EC50) of approx. 80 pmol/l) [18]. At this insulin level, gluconeogenesis is still ongoing. For half-maximal inhibition of gluconeogenesis, insulin concentrations must rise to approx. 160 pmol/l in arterial circulation. In order to stimulate glucose uptake to half maximum, insulin levels must rise to even higher levels, approx. ten times the fasting insulin concentrations (25–75% percentiles for stimulating glucose uptake approx. 350–480 pmol/l) [18]. Thus, a modest rise (doubling) of fasting insulin levels will already substantially inhibit lipolysis and promote lipogenesis while gluconeogenesis is not yet inhibited. Since such small increases of systemic insulin concentrations are enough for favoring adipogenesis, fasting and diurnal insulin levels are a determinant of obesity risk. Indeed, several data support the obesity-promoting role of insulin (for a detailed review see [18]) (Fig. 2).

These include epidemiological studies, which found high fasting insulin levels (and concomitant insulin resistance) in children and adolescents to be associated with higher weight gain in later years [19]. Studies in adults are less consistent [20]. Pharmaceutical interventions that lower insulin secretion, such as treatment with diazoxide or octreotide, led to significant body weight loss [21–23]. This fits with the observation that insulin therapy promotes weight gain [24]. One probable reason is that insulin levels in the high normal range are close to EC50 concentrations for inhibition of lipolysis [18].

In mice, modest lowering of circulating insulin concentrations by genetic manipulation of insulin genes caused resistance to weight gain despite a high-fat diet [25]. Decreasing insulin gene expression in adult mice via partial gene ablation reversed diet-induced obesity [26]. In men, the *Hph*1 “T” polymorphism in the insulin gene region was found to be associated with higher fasting insulin levels and a more rapid weight gain in obese persons [27]. A Mendelian randomization analysis showed that persons with genetically determined higher insulin secretion to oral glucose exhibited a higher body mass index (BMI) [28], supporting a causal relationship between insulin and obesity risk.

Taken together, moderate to high normal levels of insulin in metabolic healthy persons appear to be a risk factor for the development of obesity.

### Elevated insulin concentrations impair cellular functions—insulin “toxicity”

There is ample evidence that transient increases of metabolic or immune mediator levels are benign physiological responses to biochemical challenges, such as the rise of systemic glucose or cytokines following meals. However, chronic elevations of such mediators, even when modest in amplitude, are usually detrimental to cellular functions [29]. In the case of glucose, the term *glucose toxicity* was coined to describe this phenomenon [30]. Prolonged conditions of elevated glucose concentrations cause dysfunction of numerous cell types in the body, including beta cells, neurons, and the endothelium, via several pathways, including increased oxidative stress and activation of the sorbitol pathway [31–33]. As described below, there seems to be a similar detrimental outcome of long-term elevated insulin concentrations on cellular functions, a corresponding term would be *insulin toxicity*.

**Fig. 3:**
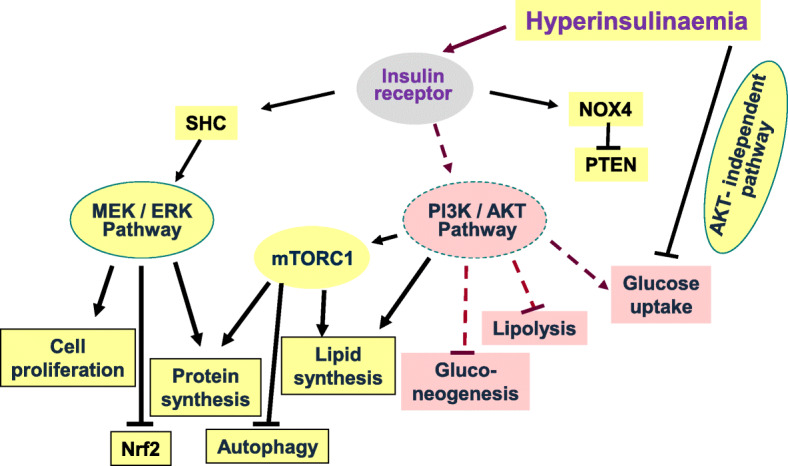
Signaling of insulin during insulin resistance. During insulin resistance, signaling through AKT kinases is partially impaired. Not all AKT-dependent pathways are affected, as well as other signaling pathways, indicating that insulin resistance is selective. Therefore, hyperinsulinemia, in the presence of insulin resistance, promotes anabolic cell activities via the MEK–ERK pathway and via mTORC1. Although the PI3K/AKT pathway is impaired during insulin resistance, and provides only insufficient translocation of GLUT4 for glucose uptake and deficient activation of eNOS, there appears to be a normal activation of mTORC1. In addition to the anabolic consequences of signaling via the MEK/ERK pathway depicted in the figure, there is enhanced expression of ET-1 and PAI-1 (not shown), as well as inhibition of autophagy and of the nuclear factor Nrf2, which compromises cell constituent turnover and cell defense mechanisms to radical stress, respectively. Hyperinsulinemia downregulates glucose uptake not only via dampening the PI3K/AKT pathway (“insulin resistance”) but also via as yet unknown other pathways

When cells are exposed to continuously elevated insulin levels, there is a partial downregulation of insulin signaling. The resulting “insulin resistance” is not primarily due to less insulin receptor expression on the cell surface but due to impaired insulin signal transduction as a result of receptor dysfunction. In response to prolonged hyperinsulinemia, there is diminished autophosphorylation of the insulin receptor, compared to that observed after short-term exposure to insulin, and subsequent steps of the PI3K–AKT signaling pathway are affected [34, 35]. Consequently, in muscle and fat cells, there is less AKT-stimulated translocation of GLUT 4 to the cell surface (Fig. 3). Thus, insulin resistance can be seen as a protective mechanism for preventing excess activation of glucose transport from the blood despite chronically elevated insulin levels, for maintaining glucose homeostasis in vivo and for mitigating metabolic and oxidative stress due to excess glucose influx [36–39]. Limiting glucose export from the blood does not necessarily require dampening of insulin signaling. During the early weeks of feeding with a high caloric diet, mice show decreased insulin-dependent glucose uptake despite unperturbed insulin-stimulated AKT phosphorylation [40, 41] (Fig. 3). An interesting aspect is that the partitioning of insulin receptor isoforms A and B and of hybrid insulin/insulin-like growth factor-1 receptors among cell types may contribute to insulin resistance in some tissues, but the pathophysiological relevance is unknown [42].

The phenomenon of insulin toxicity partly arises from the fact that there are additional cellular responses to elevated insulin levels which are not toned down during insulin resistance (Fig. 3). These comprise the upregulation of protein synthesis and the accumulation of ubiquitinated or otherwise modified proteins, probably due to insufficient degradation of these polypeptides [43]. A major role of insulin signaling via the canonical mitogen-activated protein (MAP) kinase pathway Ras–MEK–ERK, as well as via activation of NADPH oxidase 4, has been observed [4]. Even some AKT-dependent pathways do not appear to be suppressed by insulin resistance, such as de novo lipogenesis in hepatocytes or the upregulation of mechanistic target of rapamycin complex 1 (mTORC1) [44–47]. Enhanced activity of mTORC1 leads to increased protein synthesis and to deteriorated cell functions largely because of suppressed autophagy [48].

Hence, chronic exposure of cells to high ambient insulin concentrations causes an imbalance of cellular responses because of the downregulation of some insulin signaling pathways (“insulin resistance”) but not of others. The resulting functional state of cells is characterized by an unbalanced anabolic activity of insulin favoring protein synthesis while suppressing autophagy. The latter inhibits autophagic removal and turnover of proteins and lipids, which favors cell senescence [49]. In short-term experiments of exposure to high insulin levels, a protective cellular stress response is observed, the unfolded protein response, probably due to the accumulation of derivatized proteins in the absence of enough disposal. In experimentally induced or diabetes-associated chronic insulin resistance (and hyperinsulinemia), such a protective stress response of the endoplasmic reticulum to high insulin levels is diminished or absent [50].

Another activity of insulin is the suppression of transcription of the nuclear factor Nrf2 via induction of heterogeneous ribonucleoproteins F and K [51]. Nrf2 is the central regulator of the protective response of cells against oxidative and other types of electrophile stress [52]. Suppression of Nrf2 expression is expected to impair the antioxidant and cytoprotective defense capacity of cells. Insulin signaling required for Nrf2 inhibition occurs via the MAP kinase pathway and thus is not mitigated by insulin resistance [53] (Fig. 3). It therefore can be assumed that hyperinsulinemia increases the susceptibility of cells against oxidative or other electrophile stress caused by environmental insults. Prolonged exposure of cells to high insulin concentrations can therefore be regarded as toxic. Indeed, exposure to 0.5 nmol/l insulin has been found to cause DNA damage in a number of cell types, including human lymphocytes [42, 54]. At the only concentration tested (100 nmol/l), insulin impairs oxygen radical defense and sensitizes apoptosis pathways in human islets [55]. In the brain of mice, hyperinsulinemia impairs electrophysiological functions of neurons and protein turnover, causing a transition to a senescent cell state and an accompanying cognitive decline [56]. The direct toxic property of insulin deserves further study.

### Chronically elevated insulin concentrations impair body functions

#### Longevity

The above list of detrimental cellular responses to high ambient insulin concentrations suggests concomitant functional impairments at the level of the organism. This fits with the observed impact of insulin on longevity. Studies in nonvertebrate model systems such as the nematode *Caenorhabditis elegans* or the fruit fly *Drosophila melanogaster* find that moderate to high insulin activity shortens lifespan [57, 58]. A consistent finding from mouse model studies is that decreased signaling of anabolic hormones like insulin, insulin-like growth factor, or growth hormone results in a prolonged lifespan [59]. Disruption of the insulin-receptor substrate 1 gene caused insulin-resistance with defects in insulin signaling [60] and led to an extension of lifespan by 14–16% [61]. A knockout of the insulin receptor in adipose tissue of mice resulted in an 18% increase of lifespan [62]. Disruption of the Ins1 gene and one of the two mouse Ins2 alleles lowered insulin levels by 25–34% (Ins2+/− mice versus Ins2+/+ controls) in aged female mice without altering circulating insulin-like growth factor (IGF)-1 levels. These aged experimental mice exhibited lower fasting glucose, improved insulin sensitivity, and 3–11% lifespan extension across two different diets [63]. Concomitantly, the proteome and transcriptome indicated a profile associated with healthy aging. An important aspect is that this study selectively addressed insulin. Other interventions for promoting longevity or extending healthspan, such as caloric restriction, not only lower circadian insulin levels; but several additional hormones, including IGF-1, are also affected [64].

Insulin, IGF-1, and hybrid insulin/IGF-1 receptors share signaling via PI3K and AKT. The subsequent activation of the protein kinase mTORC1 is a major pathway for supporting somatic growth, protein synthesis, and fertility, while impeding autophagy and lifespan. Suppression of mTOR signaling by treatment with rapamycin prolongs life in model organisms and mice [65]. In humans, hyperinsulinemia in (pre) type 2 diabetes is associated with increased mTORC1 activity which may have a negative impact on beta cell survival, healthspan, and longevity [66]. In the Leiden Longevity Study, follow-up of nonagenarians for 10 years showed a strong association of low insulin and glucose levels with healthy aging [67].

Since both IGF-1 and insulin employ PI3K and AKT for signal transduction, it is difficult to disentangle the contribution of insulin versus IGF-1 to the modulation of longevity. In animal models, selective downregulation of circulating insulin levels improved the lifespan of mice, and in elderly persons of the Leiden Longevity Study, only insulin and glucose, but not IGF-1, consistently met all four pre-defined criteria of healthy aging [63, 67]. Therefore, it may be concluded that low circulating insulin concentrations are not only a marker of longevity but are causally involved in promoting healthspan or lifespan extension.

### Detrimental combination of hyperinsulinemia with insulin resistance

Insulin resistance is defined as an attenuated effect of insulin on blood glucose homeostasis, primarily by less efficient export of glucose from the blood into skeletal muscle, adipose, and liver tissue. Permanently elevated insulin concentrations in the blood are often considered as an attempt to overcome insulin resistance. Indeed, induction of insulin resistance by genetic disruption of insulin signaling, as well as by increased growth hormone levels or an inflammatory milieu, causes hyperinsulinemia [68–70]. The opposite causality is of more relevance. Hyperinsulinemia during insulin infusion in humans leads to systemic insulin resistance [71], while in vitro, high ambient insulin concentrations cause an increase in insulin resistance in isolated adipocytes [72]. A summary analysis of nine studies in rodents and seven trials in humans confirmed that the first detectable change in the fasting state, after feeding a high caloric diet for several days, is an increase of basal insulin concentrations, but not of blood glucose concentrations or insulin resistance [73]. Both increased secretion of insulin by ß cells and decreased insulin clearance in the liver contribute to elevated insulin levels post-meal, the latter being of primary importance in the case of carbohydrate-rich food [74].

**Fig. 4:**
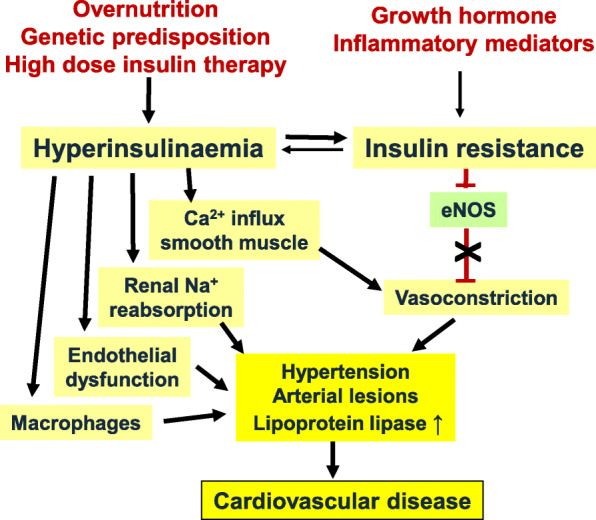
Hyperinsulinemia, insulin resistance, and cardiovascular disease. High insulin concentrations in the blood may occur due to genetic predisposition, overnutrition, or high-dose insulin treatment of type 2 diabetes. Hyperinsulinemia induces “insulin resistance” as a defense response to maintain glucose homeostasis. Conversely, insulin resistance may be directly induced such as by growth hormone or pro-inflammatory cytokines. Hyperinsulinemia and insulin resistance enhance the risk of cardiovascular disease, by inducing endothelial dysfunction, suppression of endothelial nitric oxide synthase (eNOS), and activation and promotion of calcium ion influx into smooth muscle cells, resulting in increased vascular tone, enhanced reabsorption of sodium ions in renal tubules, adhesion of macrophages to the vessel wall, and development of arterial lesions with increased lipoprotein lipase activity and cardiovascular disease

The combination of hyperinsulinemia and insulin resistance appears to promote hypertension and atherogenesis (Fig. 4). One important molecule for maintaining vessel function, including relaxation of the arterial smooth muscle layer, is nitric oxide (NO) which is generated by endothelial NO synthase (eNOS). Insulin increases NO production via posttranslational modification of eNOS via PI3K/AKT activity; however, this mechanism is suppressed during insulin resistance [75, 76]. Decreased local NO production impairs arterial smooth muscle relaxation and concomitant vasodilatation. An important factor in this context is the calcium ion homeostasis of vascular smooth muscle cells. Under physiological conditions, insulin promotes both calcium influx into the cytoplasm of smooth muscle cells via several ion channels, including L-type and store-operated Ca^2+^ channels, and counterregulatory NO-mediated efflux of Ca^2+^ and K^+^ ions which prevents calcium ion-induced myosin light chain phosphorylation and concomitant vascular contractility. During insulin resistance, NO production is impaired while the supportive effect of insulin on calcium ion influx (via PI3K delta and possibly the MEK–ERK pathway) and vasoconstriction is still present (Fig. 4) [77, 78].

At the same time, insulin signals through the mitogen-activated protein (MAP) kinase pathway to upregulate the expression of endothelin-1 (ET-1), plasminogen activator inhibitor-1 (PAI-1), adhesion molecules, and pro-inflammatory cytokines [79, 80]. The renin-angiotensin system is activated in the context of endothelial dysfunction and contributes together with decreased NO production and increased ET-1 secretion to vascular stiffening and upregulation of vascular tone [81–83]. In the absence of hyperinsulinemia/insulin resistance, the lower insulin levels exert less potential proatherogenic activities which are counteracted by insulin-stimulated local NO production [83, 84].

Elevated insulin levels also increase the risk of hypertension by enhancing renal reabsorption of sodium ions by several transport systems in different segments of the nephron (Fig. 4). Signaling of insulin occurs via insulin receptor substrate 2 (IRS2) and is not suppressed during insulin resistance, while signaling via IRS1 for counterregulatory mechanisms, including local NO production, is impaired [85, 86]. These detrimental actions may be mitigated during chronic hyperinsulinemia/insulin resistance [87]. However, a meta-analysis of 11 prospective epidemiological studies showed that the pooled relative risk of hypertension was 1.54 when comparing the highest to the lowest category of fasting insulin levels, and 1.43 for comparing highest to lowest (selective) insulin resistance categories, calculated as homeostasis model assessment of insulin resistance (HOMA-IR) [88].

As a consequence of endothelial dysfunction during prolonged treatment with insulin, arterial lesions rich in lipids are formed [89]. The progression of early fatty streak lesions to plaques is accompanied by the adhesion and pro-inflammatory activity of macrophages, which eventually develop into foam cells. This process is driven by endothelial and macrophage lipoprotein lipase activity, as demonstrated by the observation of less atherosclerosis in mice with inactivated lipoprotein lipase gene [90–92]. Lipoprotein lipase activity in macrophages is enhanced with higher insulin levels in vivo, but there is no direct stimulatory effect of insulin on isolated macrophages [93].

The concern, that hyperinsulinemia might promote arterial disease in diabetic persons, developed in the late 1960s, due to the steady increase of incidences of atherosclerosis in diabetic persons, despite improved glycemia and decreased risk of ketosis due to insulin therapy [94]. Since then, a wealth of data supports the observation that insulin resistance (and hyperinsulinemia) is a marker of increased risk of cardiovascular disease in the general population and in patients with diabetes [95]. Although observational studies suggested an approximately linear relation between the severity of hyperglycemia and vascular damage, several large randomized controlled trials have shown that intense glycemic control per se does not decrease the risk of macrovascular/cardiovascular events [96]; indeed, insulin therapy may even increase the risk [95, 97, 98]. However, these trials were not randomized for insulin treatment, and treatment of CVD risk factors was not kept similar between patient subgroups. In the United Kingdom Prospective Diabetes Study (UKPDS), hyperinsulinemia and insulin resistance were not mitigated by insulin treatment, and fasting plasma insulin levels even rose [97]. By contrast, in UKPDS and other trials [97, 99–101], oral treatment with the biguanide metformin reduced the risk of cardiovascular events and in parallel decreased insulin resistance and hyperinsulinemia.

**Fig. 5:**
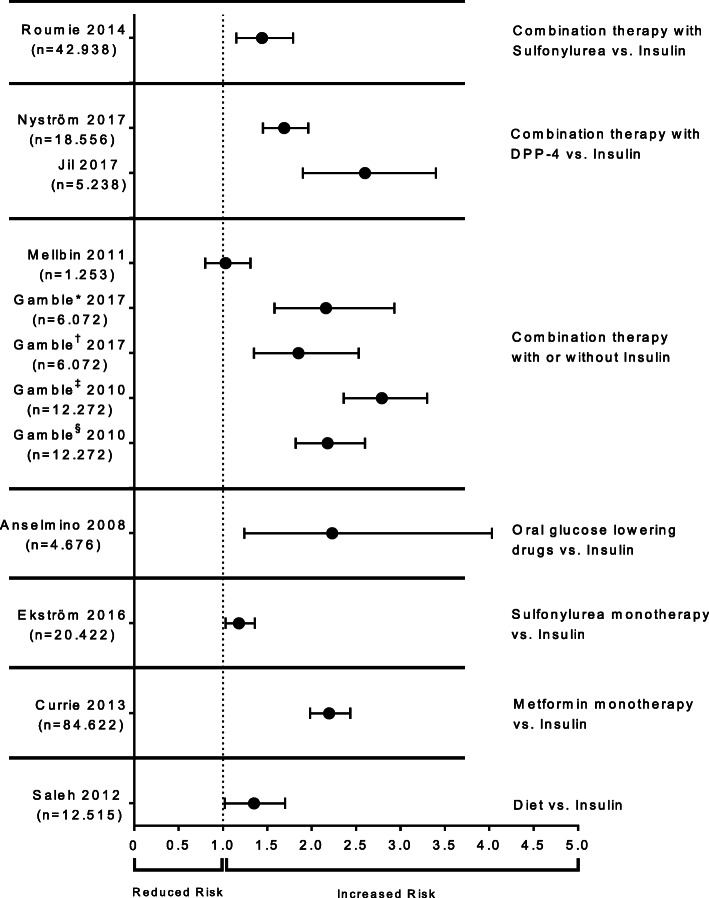
Hazard ratio of insulin medication versus different reference medications. Shown are adjusted hazard ratios (HR) for each study with 95% confidence interval. ^#^Moderate insulin exposure; ^+^high insulin exposure; *moderate insulin dose (75 to < 100 units per day); ^§^high insulin dose (> 100 units per day)

In epidemiological studies of type 2 diabetes, it has been consistently observed that the addition of insulin to the treatment regimen or the intensification of insulin treatment result in a higher rate of cardiovascular events [102–121] (Fig. 5). Indeed, it has been shown that the risk increases with increasing insulin dosage [111, 116]. These epidemiological studies may suffer from residual confounding, since it is difficult to account for the possibly more advanced disease stage of patients receiving insulin. A higher rate of hypoglycemic events may be an additional confounder. However, covariates considered in the statistical analyses cover a broad range of potential risk factors from 18 different categories (Supplement Table 1). Large randomized controlled trials such as UKPDS [122] or the Outcome Reduction With Initial Glargine Intervention (ORIGIN) Trial [123] did not observe an increased incidence of cardiovascular disease with insulin therapy, but these trials focused on low-dose insulin therapy of up to a median of 40 IU/day (or 0.4 IU/kg/day), respectively. Similar randomized trials of higher-dose insulin therapy, as typical for real-world conditions, have not been conducted. Recent studies of real-world clinical settings report mean daily basal insulin doses of close to 0.60 IU/kg in the Canadian REALITY Study for insulin-experienced patients with type 2 diabetes [124] and of 0.73 IU/kg in a physician survey in New York [125]. In the European multi-centre EU-TREAT Study, mean baseline insulin doses were between 32 and 54 U per day, depending on the type of insulin therapy regimen applied [126]. It can be concluded that under real-world conditions, the majority of insulin-experienced patients with type 2 diabetes receive higher insulin doses per day than those tried in UKPDS or ORIGIN.

In the absence of randomized controlled trials, a Mendelian randomization is an appropriate approach of testing for a causal relationship in humans. Mendelian randomization studies made use of the finding that some genotypes are associated with high or low fasting insulin levels. When comparing individuals carrying ≥ 17 alleles that raise fasting insulin levels with those exhibiting genetically determined low fasting insulin levels, an increased risk of elevated blood pressure, cardiovascular disease, and type 2 diabetes was observed [127]. In two large recent Mendelian randomization studies, a genetic profile predicting high insulin levels in the blood, after adjustment for BMI, was also associated with increased systolic blood pressure and risk of myocardial infarction [128].

## Conclusions

As discussed above, insulin signaling engages at least three different pathways and modifies a large number of cellular responses (Table 1). Transient elevations of systemic insulin concentrations are physiological responses to dietary stimuli or other challenges such as environmental toxins [129]. In case of prolonged upregulation of insulin levels, such as in response to overnutrition, glucose homeostasis is maintained by mitigating insulin signaling via PI3K/AKT for glucose export from the blood into tissues. Consequently, insulin resistance has been considered as a defense response in order to avoid hypoglycemia [38]. However, other hormonal actions of insulin via the MAP kinase MEK/ERK pathway and in part via PI3K/AKT are not or poorly inhibited by “insulin resistance.” These pathways promote a host of anabolic responses including protein synthesis. Concomitantly, there is an accumulation of ubiquinated and otherwise modified proteins. Activation of mTORC1 results in the suppression of autophagy, i.e., the removal and turnover of proteins and lipids. Signaling via MEK/ERK causes inhibition of Nrf2 activation, with the consequence of a compromised cytoprotective response to oxidative and other chemical stress. This may be the reason for increased DNA damage in the presence of high insulin concentrations. Insulin resistance suppresses the activation of eNOS by AKT, and the resulting endothelial dysfunction is enhanced by MEK/ERK-dependent expression of ET-1 and PAI-1. Further detrimental actions of insulin during insulin resistance are the promotion of calcium ion influx into smooth vascular cells favoring contractility/vascular stiffening and the enhanced sodium reabsorption in renal tubules increasing the risk of hypertension.

**Table 1:** Key messages

• Insulin employs at least three different pathways of signal transduction. One pathway involves phosphorylation steps via IRS–PI3K–AKT, another is the MAP kinases Ras–MEP–ERK, and third leads to the activation of NOX4.	
• Insulin resistance is selective because it partially mitigates the PI3K/AKT pathway for limiting glucose uptake and eNOS activation despite hyperinsulinemia, but many other hormonal actions of insulin are not suppressed.	
• Signaling via mTOR and the MEP/ERK pathway causes suppression of autophagy and NRF2, leading to deficient turnover and impaired cell defense.	
• Moderate to high normal insulin levels inhibit lipolysis and promote lipogenesis/obesity.	
• Insulin resistance and hyperinsulinemia are interdependent. Diet-induced hyperinsulinemia precedes insulin resistance.	
• In epidemiological studies, insulin therapy of type 2 diabetes is associated with a higher risk of cardiovascular events or death.	
• Randomized trials of insulin therapy and associated risks only studied dosages up to 40 IU/day.	
• Mendelian randomization studies found that genetically determined high insulin levels lead to cardiovascular disease.	
• Suppression of hyperinsulinemia and concomitant “insulin resistance” provides substantial health benefits.	

These mechanistic insights lend support to the view that the association of hyperinsulinemia with several detrimental health outcomes is of causal nature. Outcomes include obesity, endothelial dysfunction, hypertension, myocardial infarction, and decreased lifespan. We did not discuss the possible contribution of hyperinsulinemia to cancer development or to the deterioration of cognitive functions. Final proof of a causal relationship between hyperinsulinemia and disease risk cannot be obtained by randomized controlled trials, due to problems with masking the type of intervention, long-term compliance, and because of ethical concerns. Alternatively, Mendelian randomization studies are suitable tools to test for causality in humans, and such studies have found hyperinsulinemia to increase the risk of obesity [27, 28] and cardiovascular disease [127, 128].

A straightforward approach for lowering circulating insulin levels is restricting the exposure of islet ß cells to insulin secretagogues. One option is limiting calorie uptake, either continuously or during defined periods of the day or week [130–132]. Another effective way of lowering insulin levels in the blood is the stimulation of insulin clearance via exercise [133]. A different approach is a bariatric surgery [134–136]. Gastric bypass leads to rapid regression of hyperinsulinemia and later of insulin resistance; additionally, there are substantial benefits with regard to health outcomes and mortality. It seems improbable that such marked clinical improvement could have happened in the presence of persistent hyperinsulinemia and insulin resistance.

We conclude that low fasting or circadian insulin levels should be a primary aim of healthy lifestyle guidelines. Insulin treatment of type 2 diabetes seems only warranted if hyperinsulinemia and concomitant (selective) insulin resistance can be avoided. This favors insulin treatment only in the late phases of type 2 diabetes as has been suggested in recent guidelines [137].

## Supplementary information

**Additional file 1: Supplementary Table 1.** List of all regression confounders for the creation of adjusted hazard ratios.

## Data Availability

Data for this review were identified by searches from MEDLINE, PubMed, and references from relevant articles using the search terms “hyperinsulinaemia,” “insulin and longevity or Nrf2 or autophagy,” “insulin treatment and type 2 diabetes,” and “insulin signaling pathways,” In order to limit the number of references, more recently published papers referring to several previously published articles were cited, if possible. Only articles published in English were selected.

## Abbreviations

AKT: Serine/threonine-specific protein kinase B
BMI: Body mass index
DNA: Deoxyribonucleic acid
EC50: Mean insulin concentration for 50% effect
eNOS: Endothelial nitric oxide synthase
ERK: Extracellular signal-regulated kinase
ET-1: Endothelin-1
GLUT4: Glucose transporter 4
HOMA-IR: Homeostasis model assessment of insulin resistance
HR: Hazard ratio
IGF: Insulin-like growth factor
IRS: Insulin receptor substrate
MAP: Mitogen-activated protein
MEK: Mitogen-activated protein kinase kinase
mTORC1: Mechanistic target of rapamycin complex 1
NHANES: National Health and Nutrition Examination Survey
NO: Nitric oxide
NOX4: NADPH oxidase 4
Nrf2: Nuclear factor E2-related factor-2
ORIGIN: Outcome Reduction With Initial Glargine Intervention
PAI-1: Plasminogen activator inhibitor-1
PI3K: Phosphatidylinositol 3-kinase
PIP3: Phosphatidylinositol (3,4,5)-trisphosphate
PTEN: Phosphatase and tensin homolog
Raf: Rapidly accelerated fibrosarcoma
Ras: Rat sarcoma
SHC: Src homology 2 domain-containing transforming proteins
UKPDS: United Kingdom Prospective Diabetes Study
