# Supplementary material for: Insulin: too much of a good thing is bad
Source: BMC Med. 2020 Aug 21;18:224. doi: 10.1186/s12916-020-01688-6 (PMC7441661; doi:10.1186/s12916-020-01688-6)
Supplement: Supplementary file 1 — Additional file 1: Supplementary Table 1. List of all regression confounders for the creation of adjusted hazard ratios. [file 12916_2020_1688_MOESM1_ESM.docx]

**Supplementary table 1. List of all regression confounders for the creation of adjusted hazard ratios**

| **References** | **List of covariates for the adjusted hazard ratio analyses** | **Categories** |
| --- | --- | --- |
| [1] Roumie et al., 2014 | age, sex, race, fiscal year, hospitalization, months from hospitalization to intensification, nursing home use, number of outpatient visits, blood pressure, creatinine level, HbA1c, LDL, presence of proteinuria, BMI, DD, smoking, cancer, liver and respiratory failure, CHF, CVD, serious mental disease, arrythmia, COPD, asthma, HIV, Parkinson’s disease, antipsychotics, antihypertensives, antiarrhythmics, anticoagulants and platelet inhibitors, lipid lowering drugs, nitrates | 1-17 |
| [2] Nyström et al., 2017 | age, sex, DD, history of myocardial infarction, unstable angina, angina pectoris, coronary revascularization, HF, AF, stroke, TIA, PAD, major organ specific bleeding, bariatric surgery, microvascular complications, severe hypoglycemia, lower limb amputations, COPD, frailty (defined as 3 consecutive days of hospitalization the year prior to index), drugs to prevent or treat CVD (angiotensin-converting-enzyme inhibitors, angiotensin receptor blockers, beta-blockers, loop diuretics, thiazides, aldosterone, warfarin, statins, low-dose acetylsalicylic acid, antiplatelet drugs, calcium channel blockers, weight loss drugs), calendar year of both index- and first line initiation | 1, 3, 8, 9, 12, 13, 15, 17, 18 |
| [3] Jil et al.,  2017 | age, sex, social deprivation (Townsend’s index), body weight, BMI, HbA1c, total cholesterol levels, LDL, HDL, triglycerides, blood pressure, smoking, DD, GFR, albumin levels and urinary albumin–creatinine ratio | 1-7, 10 |
| [4] Mellbin et al., 2011 | age, sex, smoking, myocardial infarction, CHF, creatinine, percutaneous transluminal coronary angioplasty or coronary artery bypass grafting, mean updated blood glucose | 1, 2, 5, 8, 10, 13 |
| [5] Gamble et al., 2017 | age, sex, index of deprivation, smoking, HbA1c, chronic kidney disease stage, BMI, systolic blood pressure, number of physician visits in the year before insulin initiation, Charlson comorbidity index, CVD, duration of antidiabetic treatment, duration of metformin overlap, statins, non-steroidal anti-inflammatories, calcium channel blockers, beta-blockers, anticoagulants, antiplatelets, diuretics, agents that act on the renin-angiotensin system, antidiabetic drug therapies | 1-6, 8-13, 18 |
| [6] Gamble et al., 2010 | age, sex, chronic disease score, severity of diabetes, hospitalization in previous year, oral diabetes medications, statins, β-blockers, calcium channel blockers, angiotensin-converting enzyme inhibitors/angiotensin receptor blockers, diuretics, antiplatelets, anticoagulants, antiarrhythmics, pentoxifylline | 1, 3, 8, 9, 12, 18 |
| [7] Anselmino et al., 2008 | age, sex, previous history of coronary artery disease, previous heart failure, smoking (ever/never), fasting plasma glucose at baseline, and use at follow-up of evidence-based treatments (β-blockers, renin–angiotensin–aldosterone system blockers, oral anti-aggregants, and statins) | 1, 2, 5, 8, 9, 13 |
| [8] Ekström et al., 2016 | age, sex, diabetes duration, HbA1c, BMI, diastolic blood pressure, systolic blood pressure, total/HDL cholesterol ratio, triglycerides, microalbuminuria, GFR, smoking, physical activity, educational level, country of birth, concurrent treatment with antihypertensives, lipid-lowering agents, digoxin, anticoagulants, aspirin, organic nitrates, CVD, CHF, AF, cancer | 1-11, 13, 16 |
| [9] Currie et al., 2016 | age, sex, systolic blood pressure, HbA1c, total cholesterol, serum creatinine, BMI, smoking status, antihypertensive, lipid-lowering, antiplatelet therapy, diabetes duration, prior history of cancer, LVD, microvascular disease, number of contacts with the general practitioner in the year prior to the index date, Charlson comorbidity index. | 1-10, 12, 13, 16-18 |
| [10] Saleh et al., 2010 | age, sex, angiography indication, hospitalization, previous myocardial infarction, previous percutaneous coronary intervention, previous coronary artery bypass graft, previous HF, previous renal insufficiency and dialysis, cancer, previous stroke, previous PAD, amputation, hypertension, treatment of hyperlipidaemia, smoking, angiography findings, year of registration, retinopathy, diabetes duration, HbA1c | 1-3, 5, 8-10, 12, 13, 16-18 |

1 = baseline characteristics (age, sex)

2 = smoking

3 = diabetes history (diabetes duration (DD), disease severity, duration of antidiabetic treatment, antidiabetic drug therapies, oral diabetes medications, severe hypoglycemia)

4 = anthropometrics (body weight, body-mass-index (BMI))

5 = blood glucose (HbA1c, mean updated blood glucose)

6 = blood pressure (systolic blood pressure, diastolic blood pressure)

7 = blood lipids (triglycerides, HDL cholesterol (HDL), LDL cholesterol (LDL), total cholesterol)

8 = heart, antiplatelet and hypertensive therapy (β-blockers, calcium channel blockers, angiotensin-converting enzyme inhibitors/angiotensin receptor blockers, diuretics, concurrent treatment with antihypertensives, antihypertensives, digoxin, antiarrhythmics, nitrates, drugs to prevent or treat CVD, percutaneous transluminal coronary angioplasty or coronary artery bypass grafting, angiography indication, angiography findings, organic nitrates, previous percutaneous coronary intervention, loop diuretics, thiazides, aldosterone, anticoagulants, platelet inhibitors, low-dose acetylsalicylic acid, antiplatelet drugs, antiplatelet therapy, warfarin, non-steroidal anti-inflammatory drugs, pentoxifylline, aspirin)

9 = lipid lowering and weight loss therapy (statins, lipid-lowering agents, weight loss drugs, bariatric surgery)

10 = renal function (serum creatinine, creatinine level, microalbuminuria, albumin levels and urinary albumin–creatinine ratio, glomerular filtration rate (GFR), presence of proteinuria, chronic kidney disease stage, previous renal insufficiency and dialysis)

11 = socioeconomic variables (country of birth, race, fiscal year, physical activity, educational level, social deprivation (Townsend’s index), index of deprivation)

12 = health services variables (hospitalization, months from hospitalization to intensification, nursing home use, number of outpatient visits, frailty (defined as 3 consecutive days of hospitalization the year prior to index), number of physician visits in the year before insulin initiation, number of contacts with the general practitioner in the year prior to the index date)

13 = heart and cardiovascular diseases (congestive heart failure (CHF), cardiovascular disease (CVD), history of myocardial infarction, unstable angina, angina pectoris, coronary revascularization, heart failure (HF), atrial fibrillation (AF), stroke, transitory ischemic attack (TIA), peripheral artery disease (PAD), arrythmia, left ventricular dysfunction (LVD))

14 = mental diseases (serious mental disease, antipsychotics)

15 = lung diseases (respiratory failure, chronic obstructive pulmonary disease (COPD), asthma)

16 = cancer diseases (prior history of cancer, cancer)

17 = other diseases (HIV, Parkinson’s disease, amputation, lower limp amputation, microvascular disease, retinopathy, liver failure, hyperlipidemia, major organ specific bleeding)

18 = other variables or disease scores (year of registration, calendar year of both index- and first line initiation, Charlson comorbidity index, chronic disease score)

**References**

[1] Roumie CL, Greevy RA, Grijalva CG, Hung AM, Liu X, Murff HJ *et al*. Association between intensification of metformin treatment with insulin vs sulfonylureas and cardiovascular events and all-cause mortality among patients with diabetes. JAMA.2014;311**:**2288-96.

[2] Nystrom T, Bodegard J, Nathanson D, Thuresson M, Norhammar A, Eriksson JW. Second line initiation of insulin compared with DPP-4 inhibitors after metformin monotherapy is associated with increased risk of all-cause mortality, cardiovascular events, and severe hypoglycemia. Diabetes Res Clin Pract.2017;123**:**199-208.

[3] Jil M, Rajnikant M, Richard D, Iskandar I. The effects of dual-therapy intensification with insulin or dipeptidylpeptidase-4 inhibitor on cardiovascular events and all-cause mortality in patients with type 2 diabetes: A retrospective cohort study. Diab Vasc Dis Res.2017;14**:**295-303.

[4] Mellbin LG, Malmberg K, Norhammar A, Wedel H, Ryden L. Prognostic implications of glucose-lowering treatment in patients with acute myocardial infarction and diabetes: experiences from an extended follow-up of the Diabetes Mellitus Insulin-Glucose Infusion in Acute Myocardial Infarction (DIGAMI) 2 Study. Diabetologia.2011;54**:**1308-17.

[5] Gamble JM, Chibrikov E, Twells LK, Midodzi WK, Young SW, MacDonald D *et al*. Association of insulin dosage with mortality or major adverse cardiovascular events: a retrospective cohort study. Lancet Diabetes Endocrinol.2017;5**:**43-52.

[6] Gamble JM, Simpson SH, Eurich DT, Majumdar SR, Johnson JA. Insulin use and increased risk of mortality in type 2 diabetes: a cohort study. Diabetes Obes Metab.2010;12**:**47-53.

[7] Anselmino M, Ohrvik J, Malmberg K, Standl E, Ryden L. Glucose lowering treatment in patients with coronary artery disease is prognostically important not only in established but also in newly detected diabetes mellitus: a report from the Euro Heart Survey on Diabetes and the Heart. Eur Heart J.2008;29**:**177-84.

[8] Ekstrom N, Svensson AM, Miftaraj M, Franzen S, Zethelius B, Eliasson B *et al*. Cardiovascular safety of glucose-lowering agents as add-on medication to metformin treatment in type 2 diabetes: report from the Swedish National Diabetes Register. Diabetes Obes Metab.2016;18**:**990-8.

[9] Currie CJ, Poole CD, Evans M, Peters JR, Morgan CL. Mortality and other important diabetes-related outcomes with insulin vs other antihyperglycemic therapies in type 2 diabetes. J Clin Endocrinol Metab.2013;98**:**668-77.

[10] Saleh N, Petursson P, Lagerqvist B, Skuladottir H, Svensson A, Eliasson B *et al*. Long-term mortality in patients with type 2 diabetes undergoing coronary angiography: the impact of glucose-lowering treatment. Diabetologia.2012;55**:**2109-17.
